# Validation of the SF-6D Health State Utilities Measure in Lower Extremity Sarcoma

**DOI:** 10.1155/2014/450902

**Published:** 2014-03-19

**Authors:** Kenneth R. Gundle, Amy M. Cizik, Stephanie E. W. Punt, Ernest U. Conrad, Darin J. Davidson

**Affiliations:** Department of Orthopaedics & Sports Medicine, University of Washington Medical Center, 1959 Pacific Street NE, Box 356500, Seattle, WA 98195, USA

## Abstract

*Aim*. Health state utilities measures are preference-weighted patient-reported outcome (PRO) instruments that facilitate comparative effectiveness research. One such measure, the SF-6D, is generated from the Short Form 36 (SF-36). This report describes a psychometric evaluation of the SF-6D in a cross-sectional population of lower extremity sarcoma patients. *Methods*. Patients with lower extremity sarcoma from a prospective database who had completed the SF-36 and Toronto Extremity Salvage Score (TESS) were eligible for inclusion. Computed SF-6D health states were given preference weights based on a prior valuation. The primary outcome was correlation between the SF-6D and TESS. *Results*. In 63 pairs of surveys in a lower extremity sarcoma population, the mean preference-weighted SF-6D score was 0.59 (95% CI 0.4–0.81). The distribution of SF-6D scores approximated a normal curve (skewness = 0.11). There was a positive correlation between the SF-6D and TESS (*r* = 0.75, *P* < 0.01). Respondents who reported walking aid use had lower SF-6D scores (0.53 versus 0.61, *P* = 0.03). Five respondents underwent amputation, with lower SF-6D scores that approached significance (0.48 versus 0.6, *P* = 0.06). *Conclusions*. The SF-6D health state utilities measure demonstrated convergent validity without evidence of ceiling or floor effects. The SF-6D is a health state utilities measure suitable for further research in sarcoma patients.

## 1. Introduction

The inclusion of patient-reported outcomes (PRO) is essential to the evaluation of interventions, in order to elucidate the impact of illness and treatments on the patient experience. PRO measures of health state utilities are tools to directly elicit health-related quality of life (HRQL), and they will be pivotal in the advancement of comparative effectiveness research (CER). A recent effectiveness guidance document by the Center for Medical Technology Policy (CMTP) on incorporating patient-reported outcomes in oncology research recommends the inclusion of PRO in prospective clinical CER studies in oncology, assessment of HRQL, and use of a measure that enables cost-utility analysis [1].

The majority of PRO instruments were not designed for use in economic or value-based evaluation. Without explicitly incorporated patient preferences into the scoring algorithm, measures such as the Short-Form 36 (SF-36) [2, 3] assume equal intervals between response choices and assume that each item is of equal importance. Without an understanding of how a population values one state of health in comparison to others, the relative utility of an intervention cannot be determined. The clinical relevance of the resulting nonpreference-based scores can be challenging to ascertain.

Health state utilities are a type of PRO that merge a respondent's health status with a preference for that health state, generating a single value that facilitates comparisons among interventions, as well as disparate conditions [4, 5]. These measures provide a score ranging between 0, representing death, and 1, representing perfect health. According to utility theory, the score represents an indifference to two treatment options, one associated with maintaining the current health state and the other improving from the current state to perfect health, but also risking immediate death with a probability of 1-p, where p represents the health state score. Furthermore, health state utilities scores can be combined with time intervals to calculate quality-adjusted life years (QALYs) and enable cost-utility analyses [6–8].

Due to these capabilities, health state utility measures are gaining importance in outcomes research. One such measure, the SF-6D, may be generated from the widely utilized SF-36 quality of life PRO measure [3, 5, 9]. From the SF-36, eleven questions were selected and mapped to a six-dimensional health state classification. The dimensions are physical functioning, role limitations, social functioning, pain, mental health, and vitality; each dimension has between two and six possible levels. A total of 18,000 health states can be uniquely defined [10]. Then, using a sample of the general public who ranked and valued a subset of the possible health states via a standard gamble technique, it is possible to compute a preference-weighted value for each of the possible states [11]. These values may range between zero (worst possible state) and 1.0 (no problems in any dimension).

Health state utility measures such as the SF-6D have the potential to fulfill the CMTP recommendations as a general measure to assess HRQL and facilitate CER [12]. Before widespread use, PRO measures should demonstrate validity, reliability, responsiveness, and feasibility in the population of interest. Although health state utilities have been evaluated in many conditions and populations, to our knowledge, there has been little use in sarcoma. The purpose of this study was to evaluate the SF-6D in a population of sarcoma patients.

## 2. Patients and Methods

As part of an ongoing prospective cohort with Institutional Review Board approval, a cross-sectional sample of lower extremity sarcoma patients at an academic institution completed the SF-36 and TESS (Toronto Extremity Salvage Score) [13] between 2011 and 2012 and were eligible for inclusion. SF-6D health states were computed from the SF-36 and given preference weights based on a Bayesian modeling of a prior standard gamble valuation, as previously described [11]. Descriptive statistics evaluated possible floor or ceiling effects and skewness.

The primary outcome was the correlation between the SF-6D and the TESS, as a measure of convergent validity. A power analysis determined that 40 responses would be necessary to have an 80% chance of finding at least a 0.6 correlation. Statistical analysis was performed using Stata 11.0 (College Station, TX). Respondents also reported the use of a walking aid, and the SF-6D scores among those with and without walking aids were compared as a measure of face validity. Continuous variables were compared with a Student's *t*-test. Pearson linear regression was used to test for associations.

## 3. Results

Between 2011 and 2012, 55 patients completed 63 pairs of surveys. All patients with lower extremity sarcoma who had completed both the SF-36 and TESS were included. Patient characteristics are listed in Table 1. This heterogeneous cross-sectional sample included short- and long-term follow-up, a variety of diagnoses, and several combinations of treatment modalities. The sample included eleven patients with metastatic disease. Time from surgery included negative values, indicating participants who completed the surveys at time of diagnosis, prior to neoadjuvant treatment and surgery.

**Figure 1:**
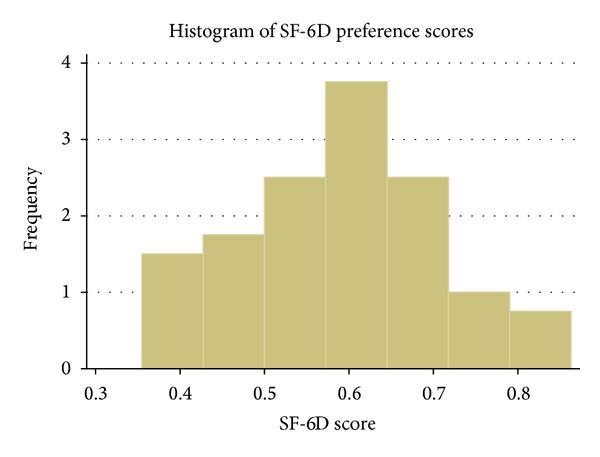
Histogram of SF-6D preference scores. Skewness = 0.11.

The mean preference weighted SF-6D score was 0.59 (95% CI 0.4–0.81). With a skewness of 0.11, the SF-6D scores closely fit a normal distribution (Figure 1). There was no significant difference in SF-6D in patients with metastatic disease (*P* = 0.88).

**Figure 2:**
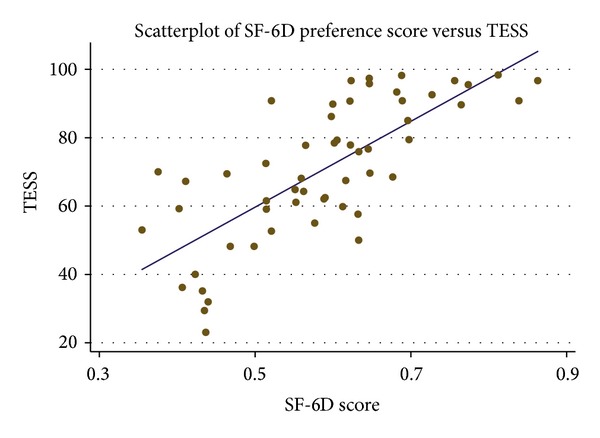
Plot of SF-6D preference scores versus TESS, with linear regression (*r* = 0.75, *P* < 0.01). TESS: Toronto Extremity Salvage Scale.

SF-6D correlated significantly with the TESS (*r* = 0.75, *P* < 0.01, Figure 2). The SF-6D correlated with the physical component scale (PCS) of the SF36 (*r* = 0.79, *P* < 0.01) as well as the mental component scale (MCS, *r* = 0.39, *P* < 0.01). While the TESS was correlated with the PCS (*r* = 0.83, *P* < 0.01), it was not significantly correlated with the MCS (*r* = 0.1, *P* = 0.44).

The SF-6D of 17 patients who reported any use of a walking aid was 0.53 (95% CI 0.48–0.59), significantly lower than those who used no ambulatory aid (*n* = 38, SF-6D = 0.61, 95% CI 0.57–0.65, *P* = 0.03). The TESS was also lower in patients reporting a walking aid (mean 59 versus 77, *P* < 0.01). The SF-6D score of 58 patients treated with limb salvage (0.6, 95% CI 0.56–0.63) was greater than the 5 patients who underwent amputation (0.48, 95% CI 0.30–0.68) but this did not achieve significance (*P* = 0.06).

## 4. Conclusions

The purpose of this study was to evaluate the validity of the SF-6D health state utility measure in a population of lower extremity sarcoma patients. Preference-based measures such as the SF-6D have the potential to facilitate comparative effectiveness research, and it is critical to establish the validity of PRO measures prior to their use in the population of interest.

In this population of lower extremity sarcoma patients, the SF-6D demonstrated convergent and face validity. The primary outcome was in correlation with the TESS, a widely used outcomes measure for extremity sarcoma. The significant positive correlation (*r* = 0.75, *P* < 0.01) between these measures is evidence of validity, as the SF-6D scores tracked appropriately across a range of TESS physical function scores. Low preference-weighted HRQL, as represented by the SF-6D results, were associated with lower physical function as represented by the TESS. And throughout the range of responses, as SF-6D scores rose so did the TESS. The TESS only assesses physical function in its content, and, unlike the SF-6D, the TESS did not correlate with the mental subscore of the SF-36. The ability to discriminate respondents with and without use of a walking aid also supports face validity of the SF-6D; this finding was convergent with the TESS. These results are consistent with the growing literature supporting the validity of the SF-6D in myriad conditions and populations [14–16].

Our finding of a close resemblance of SF-6D scores to a normal distribution in this population is important for its performance as an outcomes instrument. Significant floor or ceiling effects decrease the ability of a PRO to be sensitive to change during the course of a disease and following interventions. Previous studies have shown floor effects with the SF-6D [4, 16]. There was mild clustering at the lower end of the distribution in the present study, and patients with metastatic disease did not have a significant difference in SF-6D score. A larger sample that allows for meaningful analysis of comorbidities and burden of metastatic disease will be valuable to further assess potential floor effects of the SF-6D in this population. In contrast, the EuroQol Group's EQ-5D 3-level health state utilities measure [17] has demonstrated ceiling effects in several populations [18]. The EQ-5D has five questions, each representing a domain of health, and is scored between one and three, yielding 243 potential health states. For example, in populations with asthma or chronic obstructive pulmonary disease, over a quarter of respondents had a perfect utility score of 1.0 on the EQ-5D, while only 1 of 228 had a 1.0 utility score with the SF-6D [15]. In our study, no respondents had an SF-6D utility of 1.0, and the skewness of 0.11 reflects the near normal score distribution. High percentages of respondents scoring the top health state in the EQ-5D may also reflect insensitivity to less severe degrees of morbidity. In studies comparing these two health state utilities, there is a trend for EQ-5D scores to be higher than the SF-6D [16], and these differences can influence whether an intervention is considered cost-effective [19]. The more recently developed 5-level EQ-5D measure may be associated with fewer ceiling effects, but this has not yet been fully evaluated [20].

There are several limitations to consider. The cross-sectional, retrospective design includes a heterogeneous patient population in terms of time from surgery, type of sarcoma, and modes of treatment. This does, however, provide a sample that is representative of the different stages of treatment at which outcomes are determined. Furthermore, oncologic outcomes including recurrence and response to treatment were not assessed. While appropriate for an initial study investigating fundamental psychometric properties, no one study can establish validity. Important properties, including test-retest reliability, minimum clinically important difference, and magnitude of change, could not be established with the chosen design and require future study.

Assessing the HRQL impact of treatment decisions, such as limb salvage versus amputation, is central to the aims of reporting PRO measures. The present study had only 8% (5/63) patients treated with amputation, a subgroup too small for meaningful analysis. Further studies utilizing the SF-6D will likely contribute to this literature.

Health state utilities have the potential to facilitate comparative effectiveness research and economic modeling that incorporate patient experiences and preferences. PRO instruments with these capabilities are being recommended for all prospective oncology studies [1]. While the SF-6D can utilize the wealth of prior work and experience with the SF-36, no single health state utility measure has been convincingly proven superior [21]. This preliminary study supports the use of the SF-6D health state utilities measure in sarcoma patients, and further evaluation in a prospective cohort is warranted.

## Figures and Tables

**Table 1:** Characteristics of study participants.

Characteristic	
*n*	63
Female gender	40 (63)
Tissue type	
Bone	25 (40)
Soft tissue	38 (60)
Grade	
1	15 (24)
2	19 (30)
3	29 (46)
Surgery type	
Limb salvage	58 (92)
Amputation	5 (8)
Use of chemotherapy	34 (54)
Use of radiation therapy	37 (59)
Days from surgery	713
Mean (SD)	543 (713)
Median (range)	278 (−86–3281)

Data are presented as *n* (%) unless otherwise indicated.
